# Beyond MELD Score: Association of Machine Learning-derived CT Body Composition with 90-Day Mortality Post Transjugular Intrahepatic Portosystemic Shunt Placement

**DOI:** 10.1007/s00270-024-03886-8

**Published:** 2024-10-29

**Authors:** Tarig Elhakim, Arian Mansur, Jordan Kondo, Omar Moustafa Fathy Omar, Khalid Ahmed, Azadeh Tabari, Allison Brea, Gabriel Ndakwah, Shams Iqbal, Andrew S. Allegretti, Florian J. Fintelmann, Eric Wehrenberg-Klee, Christopher Bridge, Dania Daye

**Affiliations:** 1 Perelman School of Medicine at the University of Pennsylvania, Philadelphia, PA USA; 2 Massachusetts General Hospital, Boston, MA USA; 3 Harvard Medical School, Boston, MA USA; 4 Brigham and Womens, Boston, MA USA; 5 University of Connecticut School of Medicine, Farmington, CT USA; 6 University of Minnesota School of Medicine, Minneapolis, MN USA; 7 Tufts University School of Medicine, Boston, MA USA; 8 Massachusetts Institute of Technology, Boston, USA

**Keywords:** CT body composition, TIPS prognostication, Artificial intelligence, Machine learning

## Abstract

**Purpose:**

To determine the association of machine learning-derived CT body composition and 90-day mortality after transjugular intrahepatic portosystemic shunt (TIPS) and to assess its predictive performance as a complement to Model for End-Stage Liver Disease (MELD) score for mortality risk prediction.

**Materials and Methods:**

This retrospective multi-center cohort study included patients who underwent TIPS from 1995 to 2018 and had a contrast-enhanced CT abdomen within 9 months prior to TIPS and at least 90 days of post-procedural clinical follow-up. A machine learning algorithm extracted CT body composition metrics at L3 vertebral level including skeletal muscle area (SMA), skeletal muscle index (SMI), skeletal muscle density (SMD), subcutaneous fat area (SFA), subcutaneous fat index (SFI), visceral fat area (VFA), visceral fat index (VFI), and visceral-to-subcutaneous fat ratio (VSR). Independent *t*-tests, logistic regression models, and ROC curve analysis were utilized to assess the association of those metrics in predicting 90-day mortality.

**Results:**

A total of 122 patients (58 ± 11.8, 68% male) were included. Patients who died within 90 days of TIPS had significantly higher MELD (18.9 vs. 11.9, *p* < 0.001) and lower SMA (123 vs. 144.5, *p* = 0.002), SMI (43.7 vs. 50.5, *p* = 0.03), SFA (122.4 vs. 190.8, *p* = 0.009), SFI (44.2 vs. 66.7, *p* = 0.04), VFA (105.5 vs. 171.2, *p* = 0.003), and VFI (35.7 vs. 57.5, *p* = 0.02) compared to those who survived past 90 days. There were no significant associations between 90-day mortality and BMI (26 vs. 27.1, *p* = 0.63), SMD (30.1 vs. 31.7, *p* = 0.44), or VSR (0.97 vs. 1.03, *p* = 0.66). Multivariable logistic regression showed that SMA (OR = 0.97, *p* < 0.01), SMI (OR = 0.94, *p* = 0.03), SFA (OR = 0.99, p = 0.01), and VFA (OR = 0.99, *p* = 0.02) remained significant predictors of 90-day mortality when adjusted for MELD score. ROC curve analysis demonstrated that including SMA, SFA, and VFA improves the predictive power of MELD score in predicting 90-day mortality after TIPS (AUC, 0.84; 95% CI: 0.77, 0.91; *p* = 0.02).

**Conclusion:**

CT body composition is positively predictive of 90-day mortality after TIPS and improves the predictive performance of MELD score.

Level of Evidence: Level 3, Retrospective multi-center cohort study.

**Graphical Abstract:**

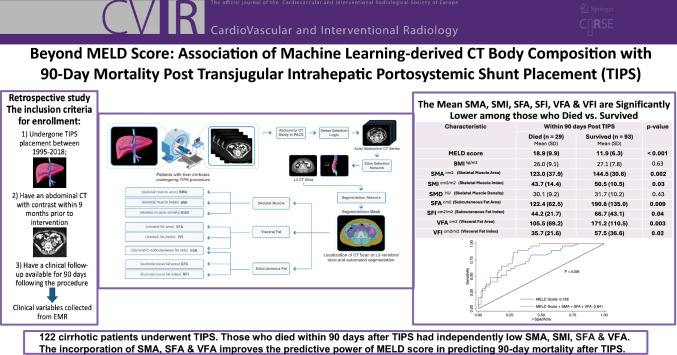

## Introduction

The loss of muscle mass and strength, termed sarcopenia, is highly prevalent in patients with cirrhosis and is associated with worse clinical outcomes [1]. However, its assessment can be challenging with body mass index (BMI), which does not account for fluid retention [2]. Many tools are proposed to assess body composition, including bioelectrical impedance analysis, hydrostatic densitometry, air displacement plethysmography, and dual-energy X-ray absorptiometry (DXA) scan [1, 3]. However, all are difficult to widely implement clinically due to their complexity, high cost, and difficulty with standardization [3]. Computed tomography (CT) scan is widely available and an established tool for body composition analysis that can risk predict and prognosticate various diseases, including nonalcoholic fatty liver [4], cirrhosis [5], cardiovascular disease [6, 7], kidney disease [8], various cancers [9–12], metabolic syndrome [13], and severe COVID-19 [14] and can predict postoperative outcomes [15–17]. Multiple machine learning algorithms and pipelines for automated analysis of CT body composition are available [18–21].

The Model for End-Stage Liver Disease (MELD) score has traditionally been used as an important predictor of mortality in cirrhotic patients, and high MELD scores are associated with early mortality after transjugular intrahepatic portosystemic shunt (TIPS) placement [22]. Studies have also shown that several CT body composition metrics are predictors of mortality in patients with cirrhosis, including those who receive TIPS placement [23]. As such, their assessment prior to the intervention might be important to predict mortality after TIPS and thus guide pre-procedural optimization for better outcomes.

In this study, our research objectives were to evaluate the association of machine learning-derived CT body composition with mortality after TIPS placement and to assess the predictive performance of body composition as a complement to the MELD score for mortality risk prediction.

## Materials and Methods

This study was approved by the Institutional Review Board at Mass General Brigham and compliant with the Health Insurance Portability and Accountability Act. The Checklist for artificial intelligence in medical imaging (CLAIM) was utilized [24].

### Study Population

**Fig. 1:**
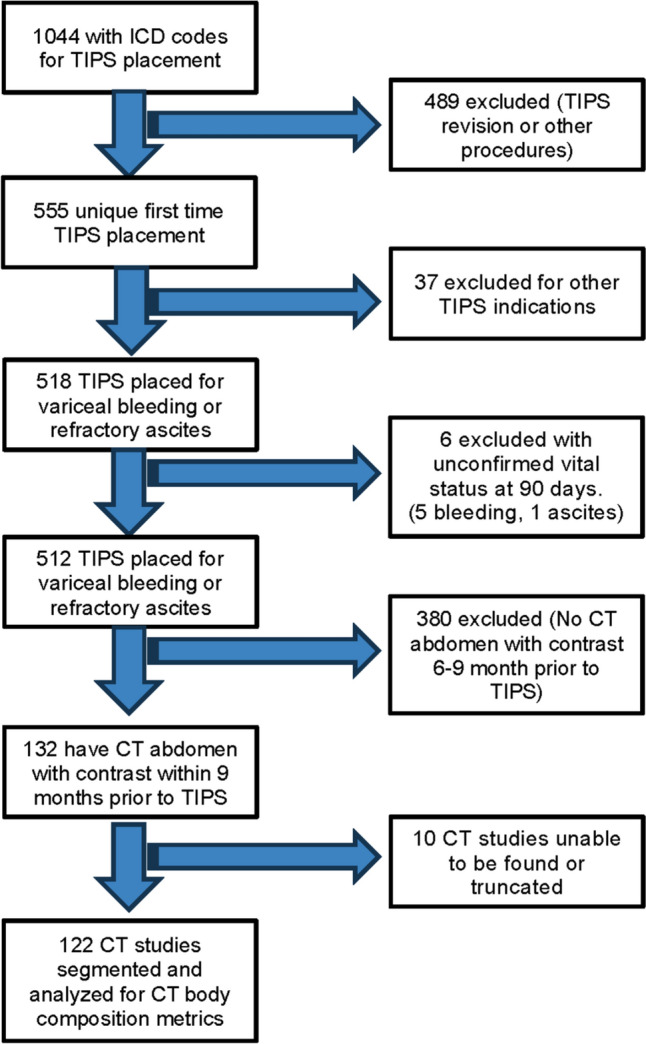
Flowchart of patient selection 122 patients met the inclusion criteria, and their CT studies were segmented and analyzed using machine learning algorithms to obtain CT body composition metrics

This was a retrospective multi-institutional cohort study done at a health-care system. Patients were identified by searching a centralized clinical data warehouse. The inclusion criteria for enrollment include: (1) TIPS placement between 1995 and 2018, (2) intravenous contrast-enhanced abdominal computed tomography (CT) scan within 9 months prior to TIPS, and (3) clinical follow-up for at least 90 days following the TIPS procedure (Fig. 1). Patients who had TIPS revisions, those without follow-up within 90 days, and those with no identifiable CT scan within 9 months prior to the TIPS procedure were excluded from the study. CT scans without contrast were excluded because the study analyzed the skeletal muscle density (SMD), a measure based on muscle attenuation which depends on the use of intravenous contrast material. Data were not readily available for 2018–up to date. Patient demographic variables, including age, sex, body mass index, weight, and height, in addition to the MELD score, were retrieved from the electronic medical record.

### Analysis of CT Body Composition

CT body composition analysis was performed using a previously validated fully automated machine learning algorithm at the L3 vertebral body level. The methodology for computing the CT body composition metrics has been described in-depth previously [25]. The analysis pipeline includes the selection of a suitable axial CT series and two convolutional neural network models (DenseNet and U-Net architectures). In the situation where a single imaging study contained multiple series suitable for analysis, the values from the series with the thickest slices, most images, and highest estimated visceral fat area (in that order of precedence) were chosen.

### CT Body Composition Metrics and Definitions

**Fig. 2:**
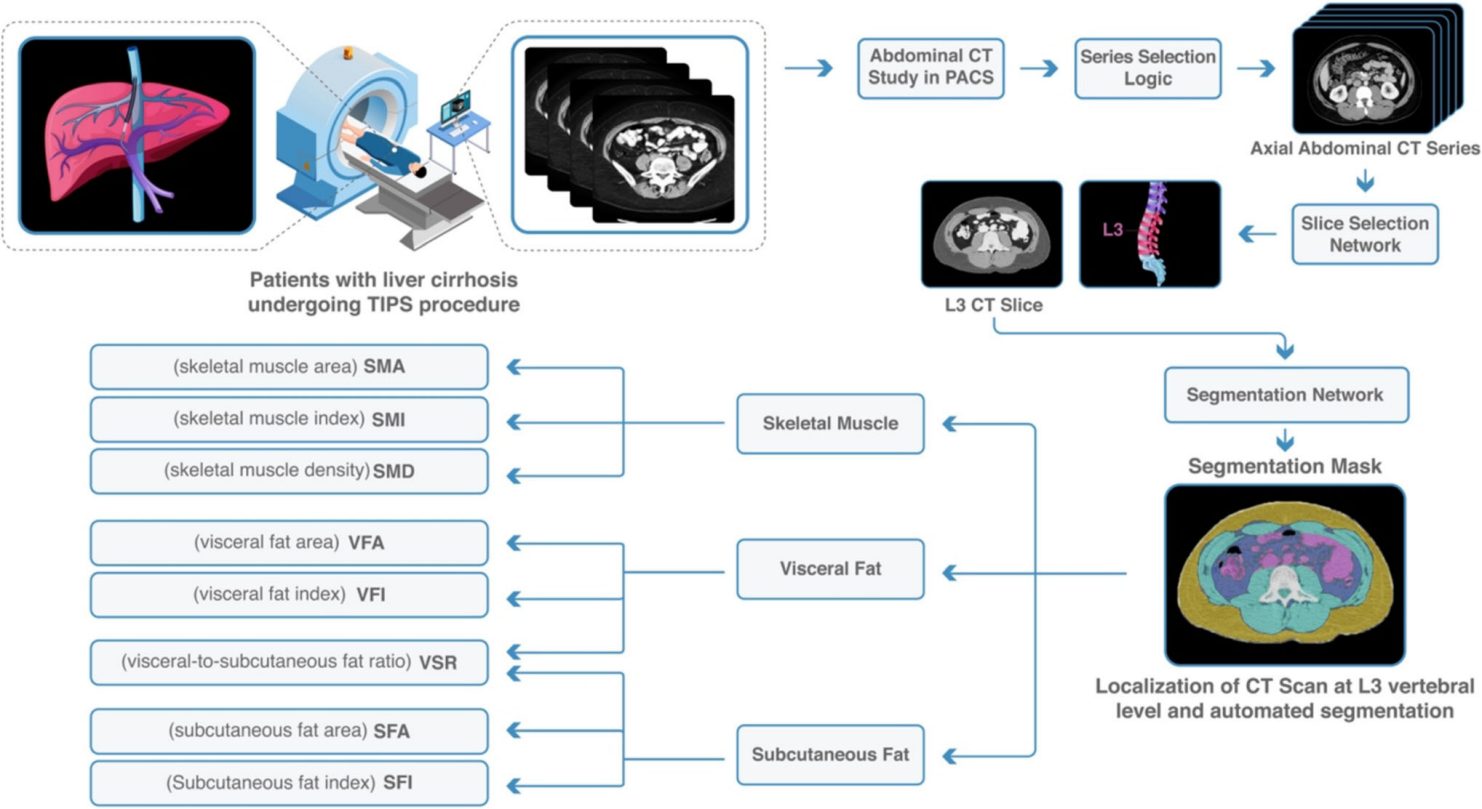
Machine learning-based CT body composition metrics. In patients undergoing TIPS procedure, automated computation of body composition from pre-procedural abdominal CT scan was performed. Body composition metrics were extracted and used to predict post-procedural outcomes. DenseNet was used for automatic L3 slice selection, and U-Net architecture model was used for segmentation

Skeletal muscle-based body composition metrics included skeletal muscle area (SMA) in cm^2^, skeletal muscle index (SMI) in cm^2^/m^2^, and skeletal muscle density (SMD) in Hounsfield units (HU). SMI was defined as SMA divided by height squared. SMD was calculated as the mean muscle radiation attenuation excluding inter- and intramuscular adipose tissue. Sarcopenia was defined as SMI < 52.4 cm^2^/m^2^ for males and SMI < 38.5 cm^2^/m^2^ for females [26]. Adipose tissue-based body composition metrics included subcutaneous fat area (SFA) in cm^2^, subcutaneous fat index (SFI) cm^2^/m^2^, visceral fat area (VFA) cm^2^, visceral fat index (VFI) cm^2^/m^2^, and visceral-to-subcutaneous fat ratio (VSR) (Fig. 2).

### Outcomes and Study Endpoint

The study endpoint was mortality within 90 days after the TIPS procedure.

### Statistical Analysis

Baseline study population characteristics were described using means and proportions overall.

The association and performance of CT body composition metrics with 90-day mortality was evaluated by comparing the SMA, SMI, SMD, SFA, SFI, VFA, VSR, and VFI of patients who survived to those who died using independent *t*-tests. MELD scores and 90-day mortality were also compared between patients who survived and those who died using independent *t*-tests.

Univariate and multivariable logistic regression models were used to compare the association between BMI and/or CT body composition metrics (SMA, SMI, SMD, SFA, SFI, VFA, VSR, and/or VFI; continuous variables) and 90-day mortality (alive vs. dead; nominal variables). Multivariable models adjusted for MELD score with each CT body composition metric individually were used to evaluate whether each metric was an independent predictor of 90-day mortality.

Receiver operating characteristic (ROC) curve analysis was employed to assess whether the inclusion of CT body composition metrics enhances the predictive efficacy of MELD in anticipating 90-day mortality after the TIPS procedure. The area under the curve (AUC) was used to assess the performance of the models. ROC curves were compared using Delong’s test to assess for statistically significant differences between models [27]. All analyses were conducted using Stata version 18 (StataCorp LP, College Station, TX). A two-tailed *p*-value < 0.05 was considered statistically significant.

## Results

### Patient Sample and Postoperative Outcomes

Between 1995 and 2018, 1044 patients were hospitalized in the health-care system with a TIPS procedure. Of these, 122 patients (11.7%) met the study inclusion criteria (Fig. 1). The patient characteristics are summarized in Table 1. Eighty-three (68%) patients were male, and 39 (32%) were female, with a mean age of 58.2 [SD ± 11.8] years. Indications for TIPS included 75 (61.5%) for variceal bleed, 53 (43.4%) for ascites, and 9 (7.4%) for hydrothorax. Among those, 15 (12.3%) had both ascites and either hydrothorax (8, 6.5%) or variceal bleed (7, 5.7%). Twenty-nine (23.8%) patients died within 90 days after TIPS. There were no associations between 90-day mortality and age (*p* = 0.36), sex (*p* = 0.90), or BMI (*p* = 0.63). Patients who died within 90 days of TIPS had higher MELD scores (18.9 vs. 11.9, *p* < 0.001) compared to those who survived, indicating the predictive power of MELD score in determining worse clinical outcomes.

**Table 1:** Characteristics of 122 patients who underwent TIPS from 2008 to 2018, stratified by 90-day mortality

Characteristic	Alive at 90 days (*n* = 93)	Dead at 90 days (*n* = 29)	*p*-value
*Age, years*			0.36
Mean (SD)	58.7 (12.5)	56.4 (9.5)	
*Sex, No. (%)*			0.90
Male	63 (67.7%)	20 (69.0%)	
Female	30 (32.3%)	9 (31.0%)	
*BMI, kg/m*^*2*^			0.63
Mean (SD)	27.1 (7.8)	26.0 (9.1)	
*MELD score*			< 0.001
Mean (SD)	11.9 (6.3)	18.9 (8.0)	

Abbreviations: BMI, body mass index; MELD, model for end-stage liver disease; and SD, standard deviation

### CT Body Composition Metrics

Detailed CT body composition metrics are presented in Table 2. Patients who died within 90 days after TIPS had a lower SMA (123 vs. 144.5, *p* = 0.002) and SMI (43.7 vs 50.5, *p* = 0.03) than those who survived. There was no difference in SMD between the two groups (30.1 vs. 31.7, *p* = 0.43). Fat-based CT body composition analysis showed that patients who died within 90 days had lower SFA (122.4 vs. 190.8, *p* = 0.009), SFI (44.2 vs. 66.7, *p* = 0.04), VFA (105.5 vs. 171.2, *p* = 0.003), and VFI (35.7 vs. 57.5, *p* = 0.02). There was no difference in VSR between the two groups (0.97 vs. 1.03, *p* = 0.66).

**Table 2:** Skeletal muscle- and fat-based CT body composition metrics of 122 patients who underwent TIPS from 2008 to 2018, stratified by 90-day mortality

Characteristic	Survived within 90 days (*n* = 93)	Died within 90 days (*n* = 29)	*p*-value
*SMA, cm*^*2*^			0.002
Mean (SD)	144.5 (30.8)	123.0 (37.9)	
*SMI, cm*^*2*^*/m*^*2*^			0.03
Mean (SD)	50.5 (10.5)	43.7 (14.4)	
*SMD, HU*			0.43
Mean (SD)	31.7 (10.2)	30.1 (9.2)	
*SFA, cm*^*2*^			0.009
Mean (SD)	190.8 (135.0)	122.4 (62.5)	
*SFI, cm*^*2*^*/m*^*2*^			0.04
Mean (SD)	66.7 (43.1)	44.2 (21.7)	
*VFA, cm*^*2*^			0.003
Mean (SD)	171.2 (110.5)	105.5 (69.2)	
*VFI, cm*^*2*^*/m*^*2*^			0.02
Mean (SD)	57.5 (36.6)	35.7 (21.6)	
*VSR*			0.66
Mean (SD)	1.03 (0.65)	0.97 (0.69)	

*SD* standard deviation; *HU* Hounsfield units; *SMA* skeletal muscle area; *SMI* skeletal muscle index; *SMD* skeletal mean density; *SFA* subcutaneous fat area; *SFI* subcutaneous fat index; *VFA* visceral fat area; *VFI* visceral fat index; and *VSR* visceral-to-subcutaneous fat ratio

### Predictive Performance of CT Body Composition Metrics

Table 3 details findings derived from both univariate and multivariable logistic regression models, elucidating the correlation between 90-day mortality and distinct body composition metrics. In the unadjusted analysis, heightened MELD scores, along with diminished SMA, SMI, SFA, VFA, and VFI scores, exhibited a significant association with 90-day mortality. Upon accounting for MELD score, the significance persisted for reduced SMA, SMI, SFA, and VFA values as robust indicators of 90-day mortality following TIPS.

**Table 3:** Univariate and multivariable regression models for 90-day mortality

Characteristic	Odds ratio	95% CI	*p*-value
*Univariate*			
SMA	0.98	0.96, 0.99	< 0.01
SMI	0.95	0.90, 0.99	0.04
SFA	0.99	0.98, 0.99	0.01
SFI	0.98	0.96, 1	0.054
VFA	0.99	0.98, 0.99	< 0.01
VFI	0.97	0.95, 0.99	0.03
MELD	1.16	1.08, 1.25	< 0.01
BMI	0.98	0.92, 1.05	0.63
Multivariate*			
SMA	0.97	0.96, 0.99	< 0.01
SMI	0.94	0.90, 0.99	0.03
SFA	0.99	0.98, 0.99	0.01
SFI	0.98	0.96, 0.99	0.049
VFA	0.99	0.98, 0.99	0.02
VFI	0.98	0.96, 1.00	0.08

^*^Each model was adjusted for MELD score

*SMA* skeletal muscle area; *SMI* skeletal muscle index; *SFA* subcutaneous fat area; *SFI* subcutaneous fat index; *VFA* visceral fat area; and *VFI* visceral fat index

### Performance with MELD Score

**Fig. 3:**
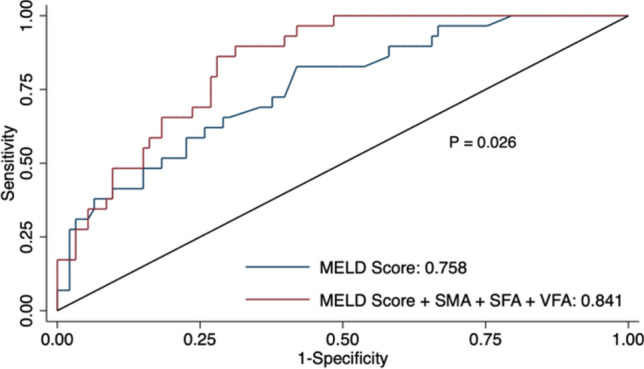
Receiver operating characteristic for prediction of 90-day mortality. Receiver operating characteristics for a model using MELD score alone (*blue line*) in predicting 90-day mortality with an AUC of 0.76 and a model using MELD score in addition to SMA, SFA, and VFA (*red line*) with AUC of 0.84 (*p* = 0.026). *SMA* skeletal muscle area; *SFA* subcutaneous fat area; and *VFA* visceral fat area

The performance of several models using MELD and body composition metrics to predict 90-day mortality is summarized in Table 4. A steady increase in AUC was noted as body composition metrics were added to the MELD score. ROC curves from multivariable logistic regressions adjusting for MELD score generated AUCs of 0.80, 0.81, and 0.77 for SFA, VFA, and VFI, respectively, with each of them maintaining independent contribution to the regression model (*p* < 0.05 on Wald test). The models that incorporated just skeletal muscle- or fat-based composition metrics to the MELD score achieved higher AUCs than the model with MELD alone, but the differences did not reach statistical significance. The AUC for predicting 90-day mortality was significantly greater in the model that incorporated both skeletal muscle- and fat-based composition metrics to the MELD score (0.84 [95% CI: 0.77–0.91] versus 0.76 [95% CI: 0.66–0.86]) and showed statistical significance (*p* = 0.02 on Delong’s test) (Fig. 3).

**Table 4:** Performance metrics of models

Model*	AUC	95% CI	*p*-value
MELD only	0.76	0.66, 0.86	Reference
MELD, SFA, and VFA	0.81	0.73, 0.89	0.10
MELD and SMA	0.82	0.75, 0.90	0.08
MELD, SMA, SFA, and VFA	0.84	0.77, 0.91	0.03

^*^The predictive performance of MELD to predict 90-day mortality after TIPS showed a steady increase with incorporation of CT body composition metrics, eventually achieving statistical significance

*SMA* skeletal muscle area; *SFA* subcutaneous fat area; and *VFA* visceral fat area

## Discussion

In this retrospective cohort study, we assessed the association between machine learning-derived CT body composition metrics and 90-day mortality after TIPS placement in patients with cirrhosis and evaluated the predictive performance of these metrics in conjunction with the MELD score. Our findings indicate that most machine learning-based CT-derived skeletal muscle- and fat-based body composition metrics are associated with 90-day mortality after TIPS and that adding CT-based composition metrics to the MELD score significantly improves the predictive power for 90-day mortality after TIPS (AUC, 0.84; 95% CI: 0.77, 0.91; *p* = 0.02). These findings emphasize the need for a more comprehensive pre-TIPS assessment of cirrhotic patients, which considers not only traditional markers such as the MELD score but also body composition metrics derived from CT scans. This may potentially be an optimal consideration in the future for patients to get CT scans prior to TIPS to improve post-procedural outcomes.

Despite BMI being the gold standard body composition method currently recommended by the CDC and being used by multiple predictive medical models [25], our study did not find any association of BMI with 90-day mortality after TIPS. Additionally, neither age nor sex had any association. Sarcopenia has been shown to be associated with worse clinical outcomes in various disease processes and is prevalent in patients with cirrhosis, but its assessment remains challenging, especially with BMI [26, 28]. Our study further confirms these results. Traditional methods for assessing body composition, such as bioelectrical impedance analysis and DXA, are not easily scalable for large-scale clinical trials and implementation. In contrast, CT-based body composition analysis is widely available and has emerged as a valuable tool for predicting and prognosticating various diseases, including cirrhotic patients undergoing TIPS as shown in our study, and as such can guide optimal treatment planning. To address the efficiency limitations of human analysis for large-scale use, the emergence of deep learning algorithms has reduced the analysis time while maintaining accuracy [29]. This could facilitate the widespread clinical investigation and implementation of CT-based body composition analysis for better prognostication leading to improved patient outcomes.

While the MELD score has been an important predictor of mortality in cirrhotic patients, studies have shown that sarcopenia and myosteatosis are also independent predictors of mortality, including in those who receive TIPS [23]. The MELD model currently does not account for these additional variables. With the use of CT-based analysis of body composition, we can identify those patients at higher risk and incorporate therapeutic interventions that can mitigate risk and improve outcomes.

Cirrhotic patients with sarcopenia tend to develop high ammonia levels leading to a higher risk of hepatic encephalopathy, especially after TIPS [30, 31]. Additionally, those patients have a higher risk of sepsis and death especially those undergoing liver resection, transplant, or transarterial chemo-embolization (TACE) for hepatocellular carcinoma [32]. Some observational studies have shown that TIPS may increase muscle mass and, as such, reverse sarcopenia, leading to lower mortality [27]. Similar to our results, Tuifua et al. demonstrated that variations in CT body composition within 3-month following TIPS procedure are associated with mortality [33]. Specifically, they found that patients with a higher baseline core muscle area (CMA) experienced reduced risk of mortality after TIPS. Furthermore, an enhancement in long-term survival was observed with a post-TIPS increase in CMA and macroscopic subcutaneous adipose tissue (mSAT), alongside a reduction in muscle adiposity index (MAI). In alignment with these findings, our research also identifies an association between baseline muscle mass and post-TIPS mortality yet fails to establish an association with muscle adiposity (SMD). In contrast, Tuifua et al. did not find an association between baseline adipose tissue measurements, particularly visceral fat, and mortality, while our analysis indicates that a lower visceral and subcutaneous fat are associated with increased 90-day mortality after TIPS.

Although, our study did not investigate some of this association but suggests that initial screening and therapeutic interventions to increase muscle mass before TIPS placement remain an important consideration to improve post-procedural outcomes. Performing TIPS may then lead to additional augmentation of muscle mass and mitigation of muscle loss leading to better outcomes. Studies suggest that despite high MELD scores, TIPS in late-stage cirrhotic patients can have favorable survival benefits [34]. Our study revealed a noteworthy connection between diminished muscle mass, subcutaneous fat, and visceral fat indices and an elevated 90-day mortality rate following TIPS, irrespective of the MELD score. This underscores the notion that, in non-emergent situations, interventions aimed at enhancing body composition metrics from lifestyle modifications such as exercise, weight gain, and appropriate diet should be considered to potentially foster favorable outcomes post-TIPS. Patients can also be enrolled in nutritional optimization programs and physical rehab to enhance their muscle mass while receiving the appropriate psychosocial support for optimal lifestyle modifications.

Our study has several limitations. The retrospective design may introduce selection biases. The number of patients included is relatively low, and the patients were drawn from a health-care system in Boston, limiting the generalizability. Additionally, the focus on 90-day mortality may not fully capture the spectrum of patient outcomes, and a longer follow-up period or assessment of other patient outcomes might provide further insights. Similarly, during a 9-month period, the body composition of a subject may change for multiple reasons that this study may not have accounted for. Our study specifically focused on elective TIPS procedures in non-emergency settings as the assessment of body composition could contribute to pre-procedural optimization making it most practical for clinical use. Therefore, our results are not generalizable to emergent settings (e.g., uncontrolled variceal bleeding). We did not investigate the cause of death to align our study with an intent-to-treat analysis, allowing for a more generalized examination of the predictive value of CT body composition in real-world clinical scenarios. Future prospective studies should be designed to provide a more in-depth analysis of the clinical role of CT body composition in predicting TIPS outcomes. There are other scores used to predict mortality after TIPS that our study did not consider, such as the Child–Pugh and Freiburg index of post-TIPS survival (FIPS) [35]. Because our study population was limited to 2018, as such we did not evaluate the most recently revised versions of MELD score such as MELD 3.0 which was introduced in 2023. Future studies can consider integrating CT body composition metrics to various scoring systems to enhance their prognostication performance in comparison with the traditional MELD score. Additionally, due to advances in technology, there may have been changes in TIPS technicalities over the years of our study inclusion that our study did not account for. Future studies should explore the integration of machine learning-based CT body composition analysis into clinical practice, examining ways to incorporate these metrics accessibly and equitably. Additional studies should also investigate non-enhanced CT scans and normalize it with contrast-enhanced CT analysis of body composition to enhance generalizability in clinical practice [36]. This would help further elucidate the potential benefits of using these techniques in cirrhotic patients and advance the development of personalized treatment plans.

## Conclusion

This study highlights the potential of machine learning-derived CT body composition analysis to improve risk prediction of patients with cirrhosis prior to TIPS placement independent of the MELD score. Those who died within 90 days after TIPS had independently lower SMA, SMI, SFA, and VFA. However, there were no significant associations between 90-day mortality and BMI, SMD, or VSR. The incorporation of SMA, SFA, and VFA improves the predictive power of MELD score in predicting 90-day mortality after TIPS. The availability of automated machine learning algorithms streamlines CT body composition analysis and supports clinical use, offering a promising approach to risk prediction and prognostication. Further studies are needed to assess the generalizability of our findings.

## Abbreviations

TIPS: Transjugular intrahepatic portosystemic shunt
MELD: Model for End-Stage Liver Disease
SMA: Skeletal muscle area
SMI: Skeletal muscle index
SMD: Skeletal mean density
SFA: Subcutaneous fat area
SFI: Subcutaneous fat index
VFA: Visceral fat area
VFI: Visceral fat index
VSR: Visceral-to-subcutaneous fat ratio
